# Clinical relevance of urine cultures in low-risk febrile infants under 3 months of age with negative urine dipsticks

**DOI:** 10.1007/s00467-026-07213-w

**Published:** 2026-02-23

**Authors:** Hannah Sjöstedt, Clara Velander, Jimmy Célind

**Affiliations:** 1 The Pediatric Emergency Department, Queen Silvia Children’s Hospital, Gothenburg, Sweden; 2 Department of Pediatrics, Institution for Clinical Sciences, The Sahlgrenska Academy, University of Gothenburg, Gothenburg, Sweden; 3 Gothenburg Emergency Medicine Research Group, The Sahlgrenska Academy, University of Gothenburg, Gothenburg, Sweden

**Keywords:** Urinary tract infections, Newborn, Fever, Urine/diagnostic use, Dipstick test, Pediatric emergency medicine, Asymptomatic bacteriuria

## Abstract

**Background:**

The clinical significance of positive urine cultures in febrile infants with negative dipsticks remains debated. The aim of this study was to investigate the extent to which negative urine dipsticks in febrile infants < 3 months of age correlate with clinically relevant urinary tract infections (UTIs).

**Methods:**

This prospective observational study included all febrile infants (< 3 months of age, temperature ≥ 38.0 °C) visiting the emergency department between October 2022 and November 2023 who had a negative urine dipstick. Infants with a prior UTI or known susceptibility to UTI were excluded. Positive cultures were considered clinically irrelevant in cases of mixed growth, growth of 1000 cfu/ml, asymptomatic bacteriuria with the same bacteria and resistance pattern within 3 months, or negative inflammatory markers together with spontaneously resolved fever without antibiotic treatment.

**Results:**

A total of 111 infants were included. The median age was 1 month, and 42 (38%) were female. Positive urine cultures were found in 44 infants (40%), of which 24 (22%) showed mixed growth. Among the remaining 20 (18%) isolated positive cultures, 12 (11%) were of insignificant growth (1000 cfu/ml), 17 (15%) had fever that resolved spontaneously without antibiotic treatment, and 8 (7%) were asymptomatic bacteriuria. No infant had a clinically relevant UTI.

**Conclusions:**

Clinically relevant UTIs are rare in febrile infants < 3 months of age without risk factors and with negative urine dipsticks. Urine cultures should not be obtained in all febrile infants with fever but should be reserved for those with an indication of UTI or serious bacterial infection.

**Graphical abstract:**

**Figure:**
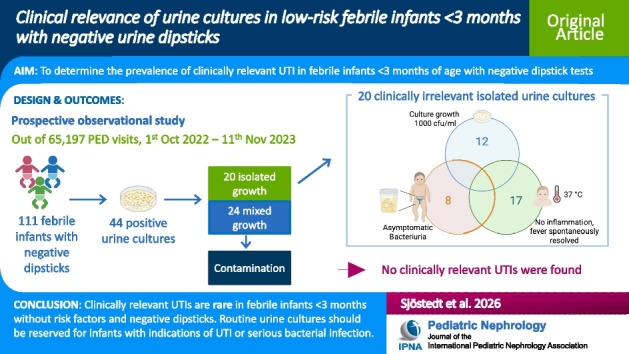
A higher resolution version of the Graphical abstract is available as Supplementary information

**Supplementary Information:**

The online version contains supplementary material available at 10.1007/s00467-026-07213-w.

## Introduction

Urinary tract infections (UTIs) are common in children, particularly during the first months of life [1]. UTI in this age group represents a potentially serious infection that may progress to sepsis or meningitis. Parents are generally advised to seek medical care if their infant presents with fever of unknown origin, even though most febrile infants do not have a serious bacterial infection [2]. Consequently, febrile infants constitute a substantial proportion of patients at pediatric emergency departments (PEDs), and screening for UTI is a central component of the diagnostic evaluation [3].

Urine dipstick analysis is a reliable screening method for the exclusion of UTI in older children and adults [1]. Its reliability in infants, however, has been debated [4–9]. According to the 2011 American Academy of Pediatrics (AAP) guidelines, the diagnosis of a clinically relevant UTI requires the presence of symptoms, pyuria, and a positive urine culture of a single bacterial strain with > 50,000 colony-forming units (cfu) per milliliter (ml) [3]. Several research groups and local guidelines have questioned these recommendations and advocate urine culture in all febrile infants regardless of urine dipstick results [8–11].

Because fever of unknown origin is common in infants < 3 months presenting to PEDs, universal urine culture testing would substantially increase workload and healthcare costs, as well as subject infants to unnecessary and painful procedures. The primary aim of the present study was to determine the prevalence of clinically relevant UTI in febrile infants under 3 months of age without precipitating risk factors of UTI with negative dipstick tests. A secondary aim was to assess whether missed febrile UTIs could have been identified by clinical or laboratory characteristics other than routine urine cultures.

## Methods

### Study population

The PED at Queen Silvia Children’s Hospital is the only PED in the Gothenburg metropolitan area, serving nine municipalities. With approximately 60,000 annual visits, it is the largest PED in the Nordic countries. All infants younger than 3 months presenting with fever between October 1, 2022, and November 11, 2023, were eligible if they had a negative dipstick test. To explore whether routine urine cultures may be unnecessary in carefully selected low-risk cases, infants with previous UTI or increased susceptibility to UTI (e.g., urinary tract reflux or other anomalies) were excluded.

At our PED, clean-catch urine is the first-line method for UTI screening. If the clinical history or symptoms suggest UTI, or if the initial sample indicates UTI, suprapubic aspiration is performed for culture. For participants in this study, who had neither a history nor a dipstick suggestive of UTI, most cultures were based on clean-catch samples.

### Study design

This was a prospective, single-center observational study. Medical chart review included data on age, sex, temperature, symptoms, systemic inflammatory markers (C-reactive protein [CRP], interleukin-6), length of hospital stay, antibiotic treatment (agent and duration), urine culture results, and follow-up within 3 months.

### Definitions

Fever was defined as ≥ 38.0 °C measured at any site (rectal, ear, axillary, or forehead), either at home or in the PED. A dipstick was defined as negative if all of the following were absent: leukocytes, nitrite, erythrocytes, protein, glucose, and ketones. Strict criteria for clinically relevant febrile UTI were applied. Cultures were deemed clinically irrelevant if any of the following were present: growth of 1000 cfu/ml, mixed bacterial growth, negative inflammatory markers combined with spontaneous resolution of fever without antibiotics, or identical bacterial growth with the same resistance pattern in an asymptomatic period within 3 months.

### Statistical analysis

Categorical variables were presented as counts and percentages. Continuous variables were presented as medians with interquartile ranges (IQR). Categorical comparisons were performed using chi-square or Fisher’s exact test, and continuous variables using the Mann–Whitney *U* test. A two-tailed *p* < 0.05 was considered statistically significant. Analyses were performed with SPSS Statistics version 29 (IBM Corp., Armonk, NY, USA, 2025). The study was approved by the Swedish Ethical Review Authority (DNR2024-05919-01), which waived the requirement for informed written consent.

## Results

### Study population

A total of 111 infants were included over the study period. Twenty-three (21%) were younger than 1 month, while 44 (40%) each were 1 or 2 months of age. Forty-two infants (38%) were female (Table 1).

**Table 1:** Characteristics of included participants (*N* = 111)

	Negative urinary cultures (*N* = 67)	Positive urinary cultures (*N* = 44)	*p* value
Age, *n* (%)			
0 months 1 month 2 months	16 (24%) 23 (34%) 28 (42%)	7 (16%) 21 (48%) 16 (36%)	0.3*
Sex, *n* (%)			
Girls Boys	26 (39%) 41 (61%)	16 (36%) 28 (64%)	0.8**
Symptoms (other than fever), *n* (%)			
Upper respiratory symptoms Vomiting Diarrhea Vomiting and diarrhea	40 (60%) 2 (3%) 0 0	23 (52%) 1 (2%) 1 (2%) 1 (2%)	0.5*
Confirmed infection, *n* (%) Otitis media Respiratory viral infection	19 (28%) 8 (12%) 11 (16%)	14 (32%) 6 (14%) 8 (18%)	0.4*
Inflammatory response, median (IQR)			
C-reactive protein† Interleukin-6	0 (0–5) 18 (10–36)	0 (0–6) 14 (5–40)	0.3*** 0.4***
Inpatient care			
Admitted, *n* (%) Days, median (IQR)	29 (43%) 1 (0.75–1.5)	17 (39%) 1 (1–1)	0.5* 0.96***
Bacterial strain (isolated cultures), *n* (%)			
*E. coli* *Enterobacteria* *Enterococcus* *S. aureus*	NA NA NA NA	14 (32%) 1 (2%) 4 (9%) 1 (2%)	

^**†**^Values 0–4 were reported as < 5 in the lab system and recorded as 0 in the data collection

^*^Pearson chi-square (2-sided)

^**^Fishers exact test (2-sided)

^***^Mann–Whitney *U* test

### Presenting symptoms

All infants presented with fever at some point. Sixty-three (57%) also had signs of viral upper respiratory tract infection, 3 (3%) had vomiting, 1 (1%) had diarrhea, and 1 (1%) had both vomiting and diarrhea.

### Urine cultures

Positive cultures were obtained from 44 infants (40%), of which 24 (22%) showed mixed growth. Among the remaining 20 (18%) isolated cultures, 14 (13%) grew *Escherichia coli*, 4 (4%) *Enterococcus*, 1 (1%) *Enterobacteriaceae*, and 1 (1%) *Staphylococcus aureus*. All isolates fulfilled criteria for being clinically irrelevant: 12 (11%) had low colony counts (1000 cfu/ml), 17 (15%) had fever that resolved without antibiotics before the urinary culture result, and 8 (7%) represented asymptomatic bacteriuria (Table 2). No clinically relevant UTI was identified.

**Table 2:** Individual results for the 8 infants with isolated bacterial growth > 1000 cfu/mL in the urinary cultures

Infant no.	Clinical characteristics				Fulfilled criteria for irrelevance	
	Bacterial strain/count	CRP/IL-6	Antibiotic treatment^1^		Asymptomatic bacteriuria^2^	Spontaneously resolved^3^
**1**	*Enteroc. *spp./10,000	7/6	1		1	
**2**	*E. coli/*10,000	6/53	1		1	
**3**	*E. coli/*10,000	< 5/23				1
**4**	*E. coli/*10,000	< 5/< 7			1	1
**5**	*E. coli/*10,000	< 5/20				1
**6**	*E. coli/*100,000	< 5/< 7	1		1	
**7**	*E. coli/*10,000	< 5/< 7				1
**8**	*Enterob.cl./*100,000	< 5/< 7			1	1

^1^Antibiotic treatment that covers urinary tract infection pathogens, not including treatment with phenoxymethylpenicillin for otitis media

^2^Renewed urinary culture in asymptomatic period within 3 months showed the same bacterial species and resistance pattern

^3^Negative serum inflammatory reactants (CRP and IL-6) and resolved fever at the result of the urinary culture without antibiotic treatment

### Antibiotic treatment

Eighty-four infants (76%) did not receive antibiotics. Of the 27 (24%) who were treated, 19 (17%) received phenoxymethylpenicillin for otitis, which does not cover typical urinary pathogens. The remaining 8 (7%) received agents active against urinary pathogens, most commonly trimethoprim–sulfamethoxazole; one infant with pneumonia was treated with cefotaxime. Three of the infants treated with agents against urinary pathogens had growth of a singular bacterial strain of significant count (Table 3).

**Table 3:** Clinical course and microbiological findings in three infants treated with antibiotics that cover urinary pathogens

Infant	Clinical course
No. 1 Boy Age 1 month	Initial urine culture: *Enterococcus spp.* (10,000 CFU/mL) in the presence of mild upper respiratory tract symptoms. CRP was 7 mg/L and IL-6 6 pg/mL. The infant was admitted for 24 h of observation with low-grade fever of < 24 h’ duration. A follow-up urine culture obtained 4 days after initial presentation, 2 days after resolution of fever, again yielded *Enterococcus spp.* (10,000 CFU/mL) with an identical resistance pattern; urine dipstick remained negative. The findings were assessed by a pediatric nephrologist as consistent with asymptomatic bacteriuria; however, a 5-day course of amoxicillin was initiated due to young age. No renal or urinary tract ultrasound was performed
No. 2 Boy Age 3 weeks	Initial urine culture: *Escherichia coli* (10,000 CFU/mL) with concurrent mild upper respiratory tract symptoms. CRP was 6 mg/L and IL-6 53 pg/mL. The infant was admitted for observation, remaining clinically well. Culture results became available the day after discharge, at which time the infant was afebrile. Repeat urine sampling 2 days later showed a negative dipstick and *E. coli* (10,000 CFU/mL) with an identical resistance pattern. A 5-day course of trimethoprim–sulfamethoxazole was initiated following pediatric nephrology consultation, despite 4 days having elapsed since resolution of fever. Renal and urinary tract ultrasound was normal
No. 6 Boy Age 2 months	Initial urine culture: *Escherichia coli* (100,000 CFU/mL) in a 2-month-old infant presenting with fever and no other clinical symptoms. CRP and IL-6 were normal, and urine dipstick was negative. The infant was managed as an outpatient and re-presented 2 days later with subfebrile temperature and irritability; repeat laboratory tests and urine dipstick remained negative. Culture results became available 4 days after sampling, at which time the infant was afebrile and feeding well. Following pediatric nephrology consultation, a 10-day course of trimethoprim–sulfamethoxazole was initiated. Renal and urinary tract ultrasound was normal. A follow-up urine culture obtained 4 weeks later during an asymptomatic period again yielded *E. coli* (10,000 CFU/mL) with an identical resistance pattern and was classified as asymptomatic bacteriuria

### Inpatient care

Thirty-six infants (32%) were admitted. Two were observed overnight in the PED without admission, and admission data were unavailable for two referred infants. Admission was more frequent among infants < 1 month (17/23, 74%) compared with those aged 1 month (23/44, 52%) and 2 months (6/42, 14%; *p* < 0.001). Median length of stay was 1 day (IQR 1–1).

### Infectious diagnoses

Thirty-three infants (30%) were diagnosed with infections other than UTI. Acute otitis media was diagnosed in 14 (13%) based on clinical examination. Nineteen (17%) had viral respiratory infections confirmed by PCR, including 8 (7%) with COVID-19, 4 (4%) with RSV, and 7 (6%) with other viruses. One case of pneumonia with rhinovirus was considered a possible secondary bacterial infection.

## Discussion

In this prospective cohort of 111 febrile infants < 3 months of age with negative urine dipsticks, positive urine cultures were common, but none was classified as clinically relevant UTI. These findings contribute to the ongoing debate balancing the need to detect serious infections against the imperative to avoid unnecessary, invasive, and potentially harmful investigations.

Our results align with the 2011 AAP guidelines, which define clinically relevant UTI as the combination of symptoms, pyuria, and a single positive culture > 50,000 cfu/ml [3]. The guidelines further state that in children 2 months to 2 years, a negative urine dipstick is a reasonable screen, allowing observation without urine culture or antimicrobial therapy. However, several studies have challenged this approach [9, 12, 13], citing reduced reliability of pyuria and nitrite in newborn infants due to frequent voiding, low dietary nitrate intake, and higher prevalence of pathogens that do not convert nitrate to nitrite [1].

A retrospective study by Lasry et al. of positive urine cultures from admitted infants ≤ 2 months of age during 11 years of study identified 2 cases of urosepsis and 32 cases of clinically relevant UTI among 308 children with positive cultures but negative dipsticks, concluding that cultures should be obtained in all febrile infants < 2 months [10]. The controversy thus centers not on whether dipsticks are imperfect but whether the rare missed UTIs would be detected clinically regardless or remain untreated with potential long-term consequences.

We anticipated identifying a small number of clinically relevant UTIs in our cohort. However, no cases were detected, preventing secondary analyses of predictors of missed UTIs. This may partly reflect our strict definition of clinical relevance, which was narrower than that used in several prior studies [5, 14]. For example, definitions based solely on symptoms and colony count (> 10,000 or > 50,000 cfu/ml) [5, 14] or combined with inflammatory markers would have classified several of our cases as UTI [10].

Clinical practice in Gothenburg favors liberal watchful waiting in well-appearing febrile infants with negative tests. This approach allowed observation of the natural course of illness in many infants without antibiotics, strengthening the validity of distinguishing clinically relevant infections from benign bacteriuria.

The key question remains how many urine cultures should be obtained to detect the few false negatives and whether these missed cases, if clinically relevant, would otherwise be identified. A multicenter study of 13,000 children by Glissmeyer et al. supports dipstick as an adequate stand-alone screen in febrile infants [5], consistent with our findings.

## Limitations

It includes the restriction to infants with negative dipsticks, precluding sensitivity and specificity analysis. Data on circumcision status were unavailable, though unlikely to influence results in a Swedish setting. The use of clean-catch specimens likely increased contamination rates, though this did not affect the primary conclusion. Strengths include the prospective population-based design, limited antibiotic exposure enabling natural disease course assessment, and detailed participant data.

## Conclusion

This prospective study demonstrates that clinically relevant UTIs are rare in febrile infants < 3 months of age without risk factors and with negative urine dipsticks. Routine urine cultures should not be obtained in all febrile infants at first presentation but reserved for those with clinical or laboratory indications of UTI or serious bacterial infection, thereby avoiding unnecessary testing, painful procedures, and inconclusive results.

## Supplementary Information

Below is the link to the electronic supplementary material.

Graphical abstract (PPTX 218 KB)

## Data Availability

Research data is not publicly available due to privacy and ethical restrictions. However, anonymized data that are minimally required to reproduce results can be made available from the corresponding author upon reasonable request, upon approval from the University of Gothenburg, if the data can be made available according to mandatory national law.
