# Supplementary material for: Clinical relevance of urine cultures in low-risk febrile infants under 3 months of age with negative urine dipsticks
Source: Pediatr Nephrol. 2026 Feb 23;41(8):2549–54. doi: 10.1007/s00467-026-07213-w (PMC13337800; doi:10.1007/s00467-026-07213-w)
Supplement: Supplementary file 1 — Graphical abstract (PPTX 218 KB) [file 467_2026_7213_MOESM1_ESM.pptx]

## Slide 1
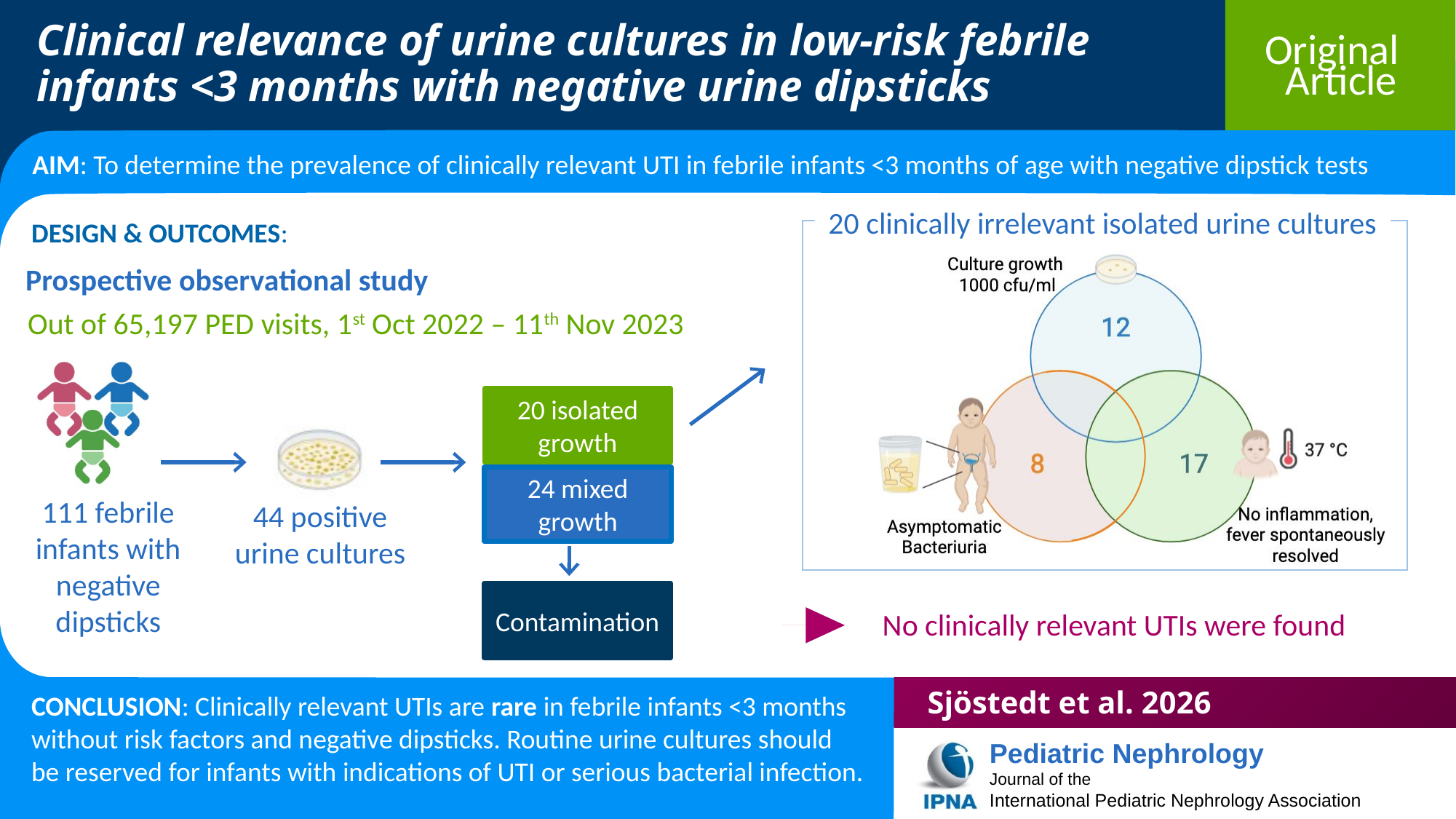

Clinical relevance of urine cultures in low-risk febrile infants <3 months with negative urine dipsticks
AIM: To determine the prevalence of clinically relevant UTI in febrile infants <3 months of age with negative dipstick tests
20 clinically irrelevant isolated urine cultures
DESIGN & OUTCOMES:
Prospective observational study
Out of 65,197 PED visits, 1st Oct 2022 – 11th Nov 2023
20 isolated growth
24 mixed growth
111 febrile infants with negative dipsticks
44 positive
urine cultures
Contamination
No clinically relevant UTIs were found
Sjöstedt et al. 2026
CONCLUSION: Clinically relevant UTIs are rare in febrile infants <3 months without risk factors and negative dipsticks. Routine urine cultures should be reserved for infants with indications of UTI or serious bacterial infection.
Prospective observational study
